# Antibacterial and Cell-Adhesive Poly(2-ethyl-2-oxazoline) Hydrogels Developed for Wound Treatment: *In Vitro* Evaluation

**DOI:** 10.1021/acs.biomac.5c00181

**Published:** 2025-04-29

**Authors:** Senem Buyuksungur, Tugba Endogan Tanir, Vasif Hasirci, Nesrin Hasirci

**Affiliations:** † Center of Excellence in Biomaterials and Tissue Engineering (BIOMATEN), Middle East Technical University (METU), Ankara 06800, Türkiye; ‡ Central Laboratory, Middle East Technical University (METU), Ankara 06800, Türkiye; § Biomedical Engineering Department, Acibadem Mehmet Ali Aydınlar University, Istanbul 34684, Türkiye; ∥ Biomaterials Center, Acibadem Mehmet Ali Aydınlar University, Istanbul 34684, Türkiye; ⊥ Department of Chemistry, Middle East Technical University (METU), Ankara 06800, Türkiye; # Department of Bioengineering, Near East University, TRNC, Mersin 10, Nicosia 99138, Türkiye

## Abstract

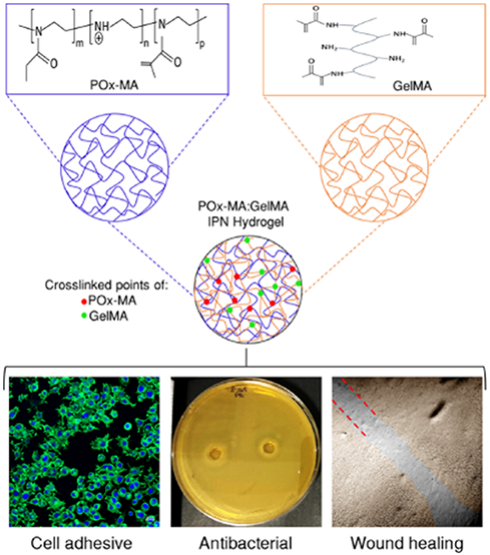

Poly(2-alkyl-2-oxazoline) (PAOx) polymers are promising materials due to their tunable properties. In this study, poly(2-ethyl-2-oxazoline) (PEtOx) was methacrylated after partial hydrolysis to produce methacrylated poly(2-ethyl-2-oxazoline) (POx-MA), which was subsequently used to synthesize novel hydrogels. Interpenetrating polymer networks (IPN) were developed by combining POx-MA with methacrylated gelatin (GelMA). Compression tests revealed that GelMA exhibited the highest mechanical strength (199 ± 21 kPa), followed by the IPN POx-MA:GelMA (112 ± 27 kPa) and POx-MA (15 ± 5 kPa). However, in scratch wound healing tests, this order was reversed, with POx-MA exhibiting the highest closure (67 ± 8%), followed by the IPN (51 ± 2%) and GelMA (42 ± 1%) in 48 h. Cell viability exceeded 90% with all of the hydrogels. The study showed that partial hydrolysis and the resultant free amine groups in POx-MA enhanced cell adhesion. Moreover, POx-MA containing hydrogels demonstrated high antibacterial activity against *Escherichia coli* and *Staphylococcus aureus*. This study highlights the superior properties of POx-MA and POx-MA:GelMA IPN as novel hydrogels with substantial potential for biomaterials and tissue engineering applications.

## 1 Introduction

Hydrogels have attracted widespread interest in the biomedical field because of their unique characteristics as three-dimensional, crosslinked polymer networks that can absorb and hold large amounts of water.^1^ In biomedical applications, hydrogels are widely used in devices, including drug delivery carriers, wound dressings, tissue engineering scaffolds, and corneal prostheses. Their biocompatibility and high capability to mimic the extracellular matrix (ECM) offer unique properties as support for cells to adhere and proliferate. Their adjustable mechanical properties and responsiveness to external stimuli, such as pH, temperature, light, and electrical or magnetic fields, provide them with additional versatility in medical applications.^2^ Natural, synthetic, or a combination of synthetic and natural polymers are used to develop hydrogels. Natural polymers, such as polysaccharides (e.g., alginate, chitosan, hyaluronic acid, dextran, cellulose, chitin) and polypeptides (e.g., collagen, gelatin, fibrin, silk fibroin, elastin), are important classes of materials because they are abundant, reasonably priced, nontoxic, and biodegradable.^3^ Biocompatibility and the presence of biologically recognizable moieties further contribute to their attractiveness in these fields. However, natural polymers often have insufficient mechanical properties for particular biomedical applications, requiring certain adjustments to align them with the needs of the application.^4^ Furthermore, when administered into the human body, they may occasionally trigger immunological or inflammatory reactions, which makes their clinical application challenging.^5^ Gelatin, which is derived from collagen, is widely preferred as a natural hydrogel source because of its biological recognition motifs like Arg-Gly-Asp (RGD) sequences which promote cell adhesion, migration, and proliferation.^6−8^ In most proteins, as well as extracellular matrix proteins, RGD motifs are the main integrin-binding domains. Cell surface integrin receptors have binding affinity for these peptide sequences, thereby mediating cellular adhesion and spreading.^9,10^ Duan et al. produced nanofibrils from *Antheraea pernyi* (Ap) silk fibers, which inherently contain RGD in their structures. These structures enhanced binding to the integrin receptors on the cell membrane and promoted fibroblast migration, adhesion, and spreading for the treatment of diabetic wounds.^11^ It was also shown that nanoparticles functionalized with RGD are highly effective in oncology, providing targeted and efficient delivery of chemotherapeutics to the tumor environment.^12^ Furthermore, the presence of targeting sequences that are sensitive to matrix metalloproteinase (MMP) facilitates cell remodeling, making it an appealing biomaterial. Gelatin can be modified chemically, such as by methacrylation, to improve its mechanical qualities, photocurability, and stability. Methacrylated gelatin (GelMA) is less immunogenic than other natural polymers, which decreases the risk of adverse immune reactions.^4,13^

On the other hand, synthetic polymers have the advantage of being easily modified and tailored into hydrogel formulations, allowing for control over physicochemical properties such as chemical composition, degradability, swelling, and mechanical properties.^14,15^ Poly(acrylamide) (PAAm), poly(vinylpyrrolidone) (PVP), poly(vinyl alcohol) (PVA), and poly(ethylene glycol) (PEG) are the most widely used synthetic polymers in the construction of hydrogels. In particular, PEG is frequently utilized in biomedical applications due to its biocompatibility, tunable mechanical properties, and capacity for functionalization with bioactive molecules.^16^ The nonfouling nature of PEG makes it a popular choice for long-term drug delivery, where PEGylated drug formulations remain in circulation for extended periods of time.^17^ It has been approved by the Food and Drug Administration (FDA) for several medical applications, especially in drug delivery as PEGylated formulations.^18^ However, potential immune reactions due to anti-PEG antibodies, the cytotoxicity of its degradation products, and limited long-term stability are serious drawbacks. In spite of these disadvantages, the broad success of PEG in biomedical applications has encouraged the development of next-generation polymeric biomaterials. These advanced materials provide increased versatility and a wider range of structural options in order to meet emerging requirements for drug loading, responsiveness, targeting, and labeling, as well as to address new medical challenges.^19^ In recent years, poly(2-alkyl-2-oxazoline) (PAOx) has been suggested as a PEG substitute because of its greater stability, customization, and functionalization.^20^ PAOx possesses the necessary qualities of biocompatibility, stealth behavior, and low dispersity. Poly(2-methyl-2-oxazoline) (PMOXA) and poly(2-ethyl-2-oxazoline) (PEtOx) are the most widely used PAOx derivatives in biomedical applications. PEtOx, which is synthesized via the cationic ring-opening polymerization (CROP) of the 2-ethyl-2-oxazoline monomer, has become a promising candidate due to its superior hydrophilicity, lack of toxicity, low immunogenicity, and stealth behavior in biological systems.^20^ Indeed, phase 1 clinical trials for the conjugated form of PEtOx with the dopamine agonist, SER-214, have been completed.^21^ PAOx-based hydrogels have recently been studied for applications such as 3D printing, injectable systems, and stem cell delivery and culture.^22,23^ Hydrogel preparation techniques using PAOx can be achieved in two ways. The first kind of crosslinking strategy involves completing the crosslinking during the polymerization process, usually by using a crosslinking agent such as 2,2’-bis(2-oxazoline) that contains two oxazoline rings.^24^ A certain disadvantage of this method is the presence of organic reagent residues in the gel network resulting from the process. The second kind of crosslinking technique involves the addition of functional groups (such as sulfhydryl, alkyne, and olefin groups) to the polymer chain, which is subsequently crosslinked by UV irradiation.^24−28^ However, all of these methods rely on complex processes, involving either complete polymerization starting from monomers or copolymerization with different monomer species. Furthermore, since PAOx is known for its cell-repellent feature, short peptides such as RGD sequences have been grafted into the polymers to obtain cell-adhesive hydrogels.^28^ There are also some studies dealing with the blending of PEtOx and GelMA, which utilized a semi-IPN structure for sciatic nerve injury regeneration and cartilage tissue regeneration.^29,30^ These studies are important as they demonstrate the positive effect of PEtOx on the physicochemical and mechanical properties and cytocompatibility of the GelMA/PEtOx hydrogels.

In this study, a novel hydrogel system was developed using methacrylated PEtOx (POx-MA), which was synthesized through sequential partial hydrolysis and methacrylation reactions and was crosslinked via UV exposure. In order to develop PEtOx-based hydrogels with long-term aqueous stability for extended applications, the incorporation of reactive functionalities is essential to enable crosslinking reactions. Photo-crosslinking offers several advantages over other chemical and physical crosslinking techniques, particularly in controlling structural integrity and mechanical properties. Consequently, methacrylate functionalization is a commonly utilized strategy for developing photo-crosslinkable hydrogels. Therefore, methacrylation was employed in our experimental design for PEtOx and gelatin in order to produce photo-crosslinkable POx-MA and GelMA, respectively. IPN hydrogels of POx-MA and GelMA were also prepared. Physicochemical and antimicrobial properties of POx-MA, IPN of POx-MA:GelMA, and GelMA were determined, and scratch wound healing tests were performed. The novel approach used in this study improved the feasibility and scalability of developing methacrylated PEtOx-based hydrogels for biomedical applications. The free amine groups formed as a result of partial hydrolysis and partial methacrylation of PEtOx in the crosslinked POx-MA hydrogel displayed antibacterial effects against *E. coli* and *S. aureus*. Furthermore, these free amine groups led to a significant level of cell adhesion by the POx-MA hydrogel. As a result, a novel hydrogel system with enhanced properties such as high cell adhesion, antimicrobial activity, and remarkable efficacy in fibroblast-mediated *in vitro* wound healing was obtained.

## 2 Materials and Methods

### 2.1 Materials

Poly(2-ethyl-2-oxazoline) (PEtOx, average Mw ∼50 kDa), porcine skin gelatin type A (90–110 bloom), methacrylic anhydride, HCl (37%), triethylamine (TEA, 99.7%), Irgacure 2959 (2-hydroxy-1-(4-(hydroxy-ethoxy)phenyl)-2-methyl-1-propanone), sodium carbonate, sodium bicarbonate, and sodium azide were purchased from Sigma-Aldrich (Germany). Paraformaldehyde (PFA) was obtained from Chemcruz (USA). Dimethyl sulfoxide (DMSO), live/dead cell viability kit, dialysis membrane (10,000 MWCO, SnakeSkin® Dialysis Tubing), and Trypsin/EDTA were obtained from Thermo Fisher Scientific (USA). The Alamar Blue kit used in cell proliferation tests was a product of Invitrogen Inc. (USA). Dulbecco’s Modified Eagle Medium (DMEM) High Glucose (glucose concentration: 4.5 g/L) and l-glutamine (200 mM in 0.85% NaCl solution) were purchased from Lonza (Switzerland). Fetal bovine serum (FBS) was a product of Biowest (France). Penicillin/streptomycin (100 U/mL to 100 μg/mL) was obtained from Fluka (Switzerland).

### 2.2 Partial Hydrolysis of PEtOx

PEtOx was partially hydrolyzed based on a procedure adapted from reported studies in the literature.^31,32^ Acidic hydrolysis of PEtOx, with an amide concentration of 0.48 M, was carried out in HCl (final acid concentration of 5.8 M) at 100 ^o^C under reflux conditions. Hydrolysis reactions were performed for 30, 60, and 120 min to obtain poly(2-ethyl-2-oxazoline-*co*-ethylene imine) (PEtOx-EI) with different ethylenimine (EI) ratios. At the end of the reaction, the solution was neutralized by the addition of sodium hydroxide pellets. Then, the solution was dialyzed (SnakeSkin, 10K MWCO, Thermo Fisher) against distilled water at 37 °C for 3 days, stored at −80 °C and freeze-dried. The lyophilized product was stored at −80 °C for further use.

### 2.3 Methacrylation of PEtOx-EI and Gelatin

PEtOx-EI was methacrylated via the reaction between the ethylenimine groups of PEtOx-EI and methacrylic anhydride, according to the method reported in the literature.^33^ Briefly, PEtOx-EI (10 %, w/v) was dissolved in a mixture of DMSO and deionized water (1:1, v/v). Methacrylic anhydride (2.5 equiv of amines) and TEA (2.5 equiv of amines) were added to the reaction mixture, and the reaction was carried out at 40 °C overnight. Then, the solution was dialyzed (SnakeSkin, 10K MWCO, ThermoFisher) against distilled water at 37 °C for 2 days, stored at −80 °C and lyophilized. The final products were stored at −80 °C for further use.

Gelatin was methacrylated by following the procedure described previously.^34^ Briefly, gelatin (20%, w/v) was dissolved in carbonate-bicarbonate (CB) buffer (0.25 M, pH 9.0). Methacrylic anhydride (MA) was added to the solution to yield a 2% (v/v) MA concentration. The reaction was performed at 50 °C for 3 h. The reaction was stopped by adjusting the pH to 7.4, and then the solution was dialyzed (SnakeSkin, 10K MWCO, ThermoFisher) against distilled water at 37 °C for 1 day, stored at −80 °C, freeze-dried and stored at +4 °C until further use.

### 2.4 ^1^H-NMR and FTIR Analysis

The degree of hydrolysis of PEtOx was determined as the percent ethylenimine (EI) conversion using a high-resolution proton nuclear magnetic resonance (^1^H-NMR) spectrometer (Bruker DPX 400) at a ^1^H resonance frequency of 400 MHz. PEtOx and PEtOx-EI with different EI conversions were dissolved in D_2_O (30 mg/mL). The degree of hydrolysis was calculated using the integrated areas of the peaks at 2.9–2.6 ppm (EI backbone) and 3.6–3.2 ppm (EOX, showing the PEtOx backbone) using the following equation:
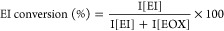
where I[EI] is the integral value of EI moieties in the backbone of the PEtOx-EI chains, and I[EOX] is the integral value of backbone PEtOx moieties.

The degree of methacrylation (DM) of PEtOx-EI-MA and GelMA was also determined by ^1^H-NMR. Samples were prepared at 30 mg/mL for analysis. The DM of PEtOx-EI-MA was calculated using the integrated areas of the peaks at 1.9–1.65 ppm (methacrylated moieties), 3.6–3.2 ppm (backbone of PEtOx groups), and 2.9–2.6 ppm (EI backbone) using the following equation:
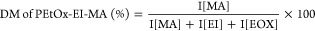
where I[MA] is the integral value of methacrylated moieties, I[EI] is the integral value of EI moieties, and I[EOX] is the integral value of PEtOx moieties.

The DM of GelMA was calculated using the integrated areas of the peaks between 2.95 and 2.8 ppm (lysine methylene) of gelatin and GelMA, using the following equation:
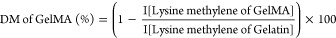


Integrated areas of the peaks were calculated using the MestreNova NMR analysis program (version 6.0.2, Mestrelab Research, S.L., Spain).

Chemical analyses of PEtOx, PEtOx-EI, and PEtOx-EI-MA were conducted by Attenuated Total Reflectance FTIR (ATR FTIR, PerkinElmer, USA) to confirm the synthesis. Likewise, gelatin was analyzed using ATR FTIR both before and after the methacrylation reaction.

### 2.5 Preparation of Hydrogels

In this study, partially hydrolyzed PEtOx with varying EI conversions (PEtOx-EI) were obtained based on three different hydrolysis reaction times (30, 60, and 120 min). Since methacrylation occurred through these EI groups, degree of methacrylation (DM) also varied with the EI conversion. Therefore, three different PEtOx-EI-MA polymers were obtained through these sequential reactions (Table 1). Among them, the ones hydrolyzed for 120 min, which have the highest EI conversion (70%) and DM (36%) (coded as PEtOx-EI_70_-MA_36_), were chosen for the preparation of PEtOx hydrogels and were defined as POx-MA hydrogel in the rest of the article.

**Table 1:** Degree of Hydrolysis and Methacrylation of PEtOx Polymer Depending on the Hydrolysis Reaction Time

Polymer	Hydrolysis Reaction Time (min)	Degree of Hydrolysis (%)	Degree of Methacrylation (%)
PEtOx			
PEtOx-EI_12_-MA_10_	30	12	10
PEtOx-EI_32_-MA_17_	60	32	17
PEtOx-EI_70_-MA_36_ (POx-MA)	120	70	36

POx-MA (15%, w/v) and GelMA (15%, w/v) solutions were prepared by dissolving them separately in phosphate-buffered saline (PBS, 10 mM, pH 7.4) containing Irgacure 2959 (1%, w/v). POx-MA:GelMA hydrogels were prepared by mixing the POx-MA and GelMA prepolymer solutions at a volume ratio of 1:1. The prepolymer solutions (100 μL) were poured into polydimethylsiloxane (PDMS) molds (5 mm diameter, 1.5 mm height) and exposed to UV light (OmniCure, S2000, 365 nm, 15 W/cm^2^, 3 cm distance) for 2 min to obtain crosslinked hydrogels. Disk-shaped gels (5 mm diameter, 1.5 mm height) were used for *in situ* characterization, *in vitro* cell culture, and antibacterial tests. Samples with a 5 mm diameter and 4 mm height were also prepared for compressive mechanical tests.

### 2.6 Morphological Analysis

POx-MA, POx-MA:GelMA, and GelMA hydrogels were examined with a scanning electron microscope (SEM, FEI Quanta 400F, The Netherlands). The lyophilized hydrogels were frozen in liquid nitrogen and cut to examine the porous structure in the cross-section. The samples were then coated with gold–palladium (Au–Pd) under argon atmosphere. The diameters of the pores in the hydrogels were measured using ImageJ NIH software (USA). At least 50 pores per image were measured, and the pore size distribution of the hydrogels was plotted as frequency (%) vs pore size (μm).

### 2.7 Swelling Properties

The swelling properties of the hydrogels were determined by measuring the water content (%) and swelling degree (SD). Before analysis, samples were lyophilized, and the initial dry weights of the samples were recorded. Then, the samples were incubated in 3 mL of distilled water at 37 °C and their weights were measured at specific time intervals (1, 3, 5, 24, and 48 h) until the samples reached their equilibrium (maximum) weight. Water contents and swelling degrees were calculated by using the following equations:
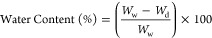

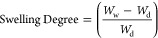
where *W*_w_ is the wet weight and *W*_d_ is the dry weight.

Equilibrium water content (EWC) and maximum swelling degree were calculated after 48 h.

### 2.8 *In Situ* Degradation Tests

The stability of hydrogels in an aqueous environment was determined by incubating them in PBS (10 mM, pH 7.4) containing 0.2% (w/v) sodium azide at 37 °C. At the beginning of the experiments, samples were lyophilized, and the initial dry weights of the samples were recorded. Incubation continued for 21 days, and at predetermined time points (on days 1, 7, 14, and 21), samples were washed with distilled water three times, lyophilized, and weighed. The remaining weight was calculated using the following equation:
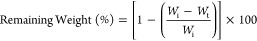
where *W*_i_ is the initial weight of the dried samples, and *W*_t_ is the weight of lyophilized samples at certain time points.

### 2.9 Mechanical Properties

Mechanical properties of the hydrogels were analyzed with a compressive mechanical test using a Mechanical Tester (CellScale Univert, Canada) equipped with a 10 N load cell. For the mechanical analysis, samples 4 mm in height were prepared as mentioned in Section 2.5. The compression test was conducted at a displacement rate of 1 mm/min. The compressive moduli of the samples were calculated from the slope of the linear region between 20% and 40% strain on the stress–strain graph.

### 2.10 *In Vitro* Cell Culture Studies

Cell culture studies were conducted with L929 fibroblasts by seeding them on the hydrogels. For cell culture studies, hydrogels (5 mm diameter and 1.5 mm height) were prepared in DMEM high-glucose media instead of PBS. The rest of the procedure for hydrogel preparation was the same as described in Section 2.5. L929 fibroblasts were seeded onto the hydrogels at an initial cell seeding density of 3x10^4^ cells/sample. Cell-seeded samples were incubated in complete media composed of DMEM high-glucose supplemented with FBS (10%, v/v) and Penicillin/Streptomycin (1%, v/v) for 21 days in a CO_2_ incubator (37 °C, 5% CO_2_) with media changes every 2 days.

#### 2.10.1 Live/Dead Staining

Viability and adherence of the cells on the hydrogels were studied with Live/Dead staining on days 1, 7, 14, and 21 using a confocal laser scanning microscope (CLSM, Zeiss LSM 800, Germany). Before analysis, the cell culture media were discarded from the wells, and the samples were washed with PBS (10 mM, pH 7.4). A dye solution containing 2 μM Calcein AM and 4 μM Ethidium homodimer-1 was added to each well, and the mixture was incubated at room temperature for 15 min. The samples were washed with PBS twice, and the cells on the hydrogels were examined under CLSM. Micrographs were analyzed using ImageJ NIH software, and cell viability (%) was calculated using the following equation:
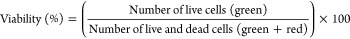


#### 2.10.2 Alamar Blue Cell Proliferation Test

Cell proliferation analysis was conducted with the Alamar Blue cell proliferation assay on days 1, 7, 14, and 21. At each time point, the cell culture media were discarded, and the samples were washed once with colorless DMEM (without phenol red). Then, the Alamar Blue solution (89% colorless DMEM colorless, 10% Alamar Blue reagent, 1% Penicillin/Streptomycin) was added, and the samples were incubated at CO_2_ incubator (37 °C, 5% CO_2_) for 1 h. The fluorescence intensity was measured at an excitation wavelength of 575 nm and an emission wavelength of 595 nm.

#### 2.10.3 Morphological Analysis

Cell morphology on the hydrogels was assessed following DAPI/Phalloidin staining of cells fixed for 15 min with a 4% paraformaldehyde (PFA) solution on day 21. Cell nuclei were stained with DAPI at room temperature for 15 min, and actin filaments were stained with Alexa Fluor 488 Phalloidin for 1 h at 37 °C. Cells were analyzed under CLSM.

#### 2.10.4 *In Vitro* Wound Healing Scratch Assay

*In vitro* wound scratch assay was carried out to study cell migration on the hydrogels, which is a key factor for wound closure. L929 fibroblasts were seeded at a density of 3 × 10^4^ cells/sample and incubated in complete media until the cells reached 80–100% confluence. One day before the test, cells were incubated in serum-free medium overnight. Then, cells were scratched from the surface using a sterile 200 μL pipette tip. Displaced cell fragments were washed with PBS, and fresh serum-free cell culture media were added to the wells. In order to verify that wound closure resulted directly from cell migration rather than cell proliferation, experiments were conducted using serum-free media. The scratched area was imaged on days 0, 1, and 2 using the ESID module of CLSM. The closure of the scratched region was quantified using the wound healing size plugin in ImageJ NIH software.^35^ The extent of wound closure was calculated according to the following equation:
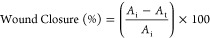
where *A*_i_ is the wound area at the beginning, and *A*_t_ is the wound area at time t, both in μm.^2^

### 2.11 Antimicrobial Tests

The zone of inhibition test was carried out to evaluate the antibacterial properties of the hydrogels using strains of Gram-negative *E. coli* (ATCC 25922) and Gram-positive *S. aureus* (ATCC 29213). Brain Heart Infusion (BHI) agar plates were seeded with overnight cultures of *E. coli* and *S. aureus* that had been adjusted to 0.5 McFarland turbidity standards.^36^ After that, the hydrogels (5 mm in diameter and 1.5 mm in height) were placed onto the agar plates and left to incubate for 24 h at 37 °C. PEtOx-EI was also evaluated for comparison with the hydrogels. Since PEtOx-EI does not have any methacrylated groups, it cannot be cross-linked; thus, it was in liquid form. Therefore, the antibacterial property of PEtOx-EI was assessed with a well diffusion method in agar plates. Wells (5 mm in diameter) were created by using a sterile punch. Then, 100 μL of PEtOx-EI, which is an equal volume to the prepolymer solutions of the hydrogels, was added into these wells, and the agar plates were left to incubate for 24 h at 37 °C. ImageJ NIH software was used to measure and analyze the zones of inhibition surrounding the hydrogels. Before the tests, all of the hydrogels and PEtOx-EI were sterilized under UV-C (265 nm) for 15 min.

### 2.12 Statistical Analysis

All *in situ* and *in vitro* tests, other than mechanical analysis, were conducted in triplicate (*n* = 3). Compressive mechanical tests were performed with five repetitions (*n* = 5). Results are expressed as mean ± standard deviation. Statistical analyses were carried out using GraphPad Prism version 8.2.1 (GraphPad Software, San Diego, CA, USA). One-way ANOVA with Tukey’s post hoc test and/or two-way ANOVA were applied depending on the number of comparisons analyzed. *p*-values ≤ 0.05 were reported as statistically significant.

## 3 Results and Discussion

### 3.1 ^1^H-NMR and FTIR Analysis

**Figure 1:**
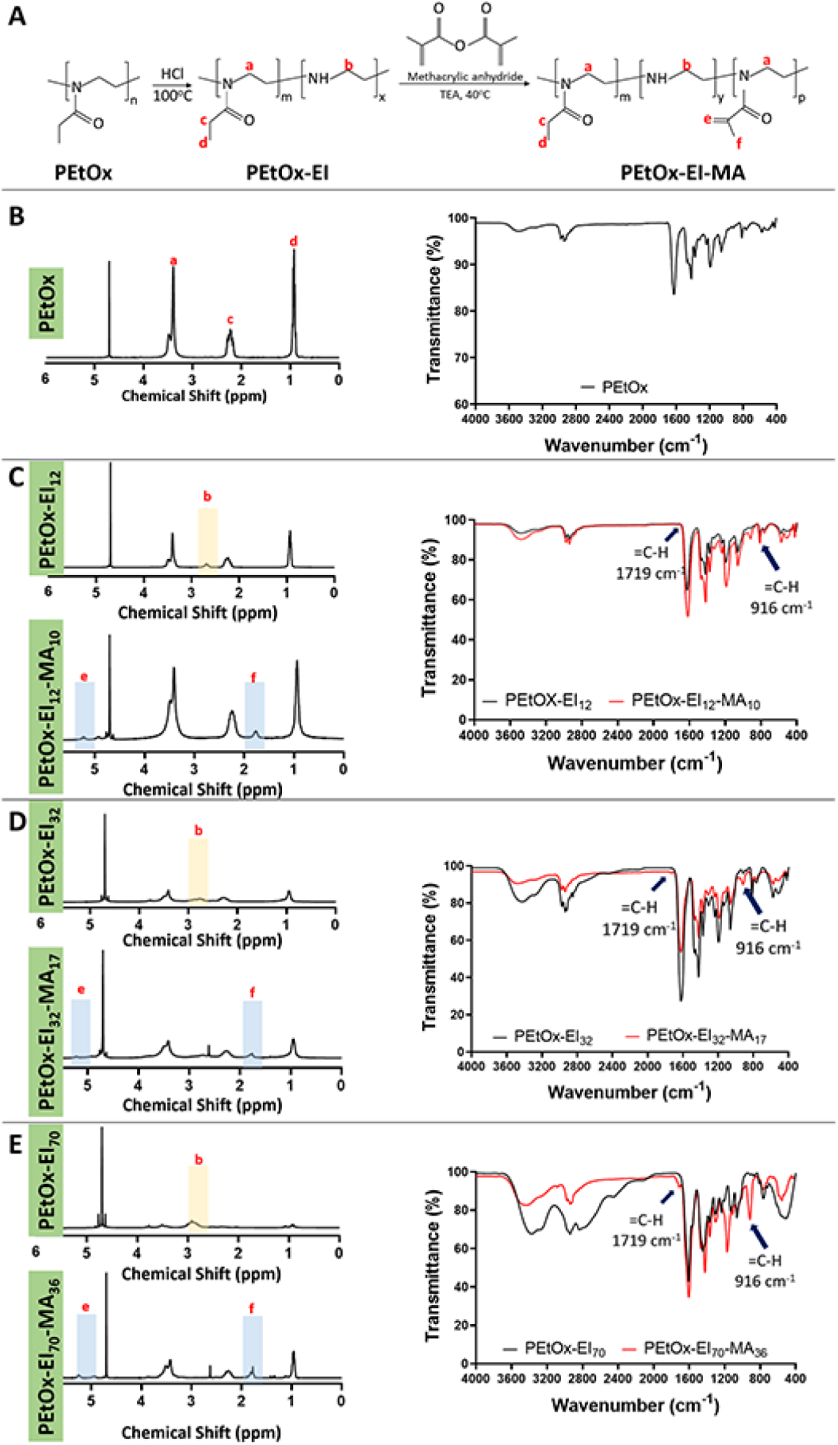
Synthesis and chemical characterization of PEtOx before and after partial hydrolysis and methacrylation reactions. (A) Sequential reactions of partial hydrolysis and methacrylation of PEtOx. ^1^H-NMR and FTIR spectra of (B) PEtOx, (C) PEtOx-EI_12_ and PEtOx-EI_12_-MA_10_, (D) PEtOx-EI_32_ and PEtOx-EI_32_-MA_17_, and (E) PEtOx-EI_70_ and PEtOx-EI_70_-MA_36_ (n, m, x, y, and p represent the number of the repeating units in the polymer molecule. n: number of original repeating units of PEtOx before the partial hydrolysis reaction (n = m + y + p); x: number of newly formed ethylenimine (EI) units after hydrolysis (x = n – m); m: number of remaining oxazoline (EOX) units that were not hydrolyzed (m = n – x); p: number of methacrylated EI units (p = x – y); y: number of unreacted (remaining) EI units (y = x – p); m: (unchanged): number of remaining oxazoline (EOX) units that were not hydrolyzed (m = n – x)).

PEtOx was modified through sequential reactions of partial hydrolysis and methacrylation to obtain polymers with methacrylated double bonds (Figure 1A). The hydrolysis kinetics of PEtOx is mainly dependent on acid concentration, temperature, and reaction time.^32,37^ In this study, the temperature and HCl concentration were kept constant at 100 °C and 5.8 M, respectively. The partial hydrolysis reaction was carried out with different reaction times (30, 60, and 120 min) to obtain different degrees of EI conversions. The ^1^H-NMR spectra of PEtOx and partially hydrolyzed PEtOx (PEtOx-EI) were used to assess the degree of EI conversions.

The PEtOx backbone was detected between 3.6 and 3.3 ppm (Figure 1B, peak a), while the PEtOx side chains were identified between 2.4 and 2.1 ppm (Figure 1B, peak c) and between 1.0 and 0.8 ppm (Figure 1B, peak d). Partial hydrolysis of PEtOx was confirmed with the reduced signals of the PEtOx backbone and the increased signal at 2.9 ppm (Figure 1C–E, peak b), which was ascribed to EI moieties. The signal attributed to EI moieties at 2.9 ppm increased and broadened as the reaction time increased (Figure 1C–E, peak b). The degree of EI conversions (%) was calculated as 12%, 32%, and 70% for reaction times of 30, 60, and 120 min, respectively. The ^1^H-NMR spectra of the methacrylated polymer, PEtOx-EI-MA, displayed signals corresponding to the double bond between 5.5 and 5.0 ppm (Figure 1C, peak e) and the methyl group of the methacryloyl groups between 1.9 and 1.65 ppm (Figure 1C, peak f). As methacrylation increased, peaks “e” and “f” became significantly larger. The secondary amines in PEtOx-EI provide reactive sites for further methacrylation of the polymer through a reaction with methacrylic anhydride. Thus, more secondary amines in the polymer result in a higher degree of methacrylation (DM). DM values were found to be 10%, 17%, and 36% for the partially hydrolyzed polymers having EI conversions of 12%, 32%, and 70%, respectively. Reaction time dependent increase in the degree of EI conversions (%) and the DM (%) is also shown in Table 1.

It is known that steric hindrance prevents the methacryloyl groups from fully substituting the secondary amines, as demonstrated by residual EI units of PEtOx-EI-MA.^33^ In line with our results, Shan et al. produced PEtOx-EI-MA polymers to increase the mucoadhesive properties of the polymer for nasal drug delivery applications.^33^ In our study, methacrylated PEtOx polymer was further cross-linked via UV, creating a hydrogel whose potential for biomedical applications, especially for wound healing, was investigated.

The infrared analysis of PEtOx revealed peaks at 1235 cm^–1^ (C—N stretch), 1470 cm^–1^ (C—H bending), 1418 cm^–1^ (C—H bending), 1626 cm^–1^ (C=O stretch), and 2975 and 2937 cm^–1^ (CH_2_ stretch). As reported by Shan et al., the characteristic N—H bending pattern of EI at 1474 cm^–1^ overlaps with the C—H bending mode of backbone moieties at 1470 cm^–1^, making it difficult to detect EI moieties in FTIR spectra.^33^ As methacrylation increased, new peaks appeared at 1719 and 916 cm^–1^, corresponding to the stretching and bending modes of =C—H, further confirming its effective modification.

**Figure 2:**
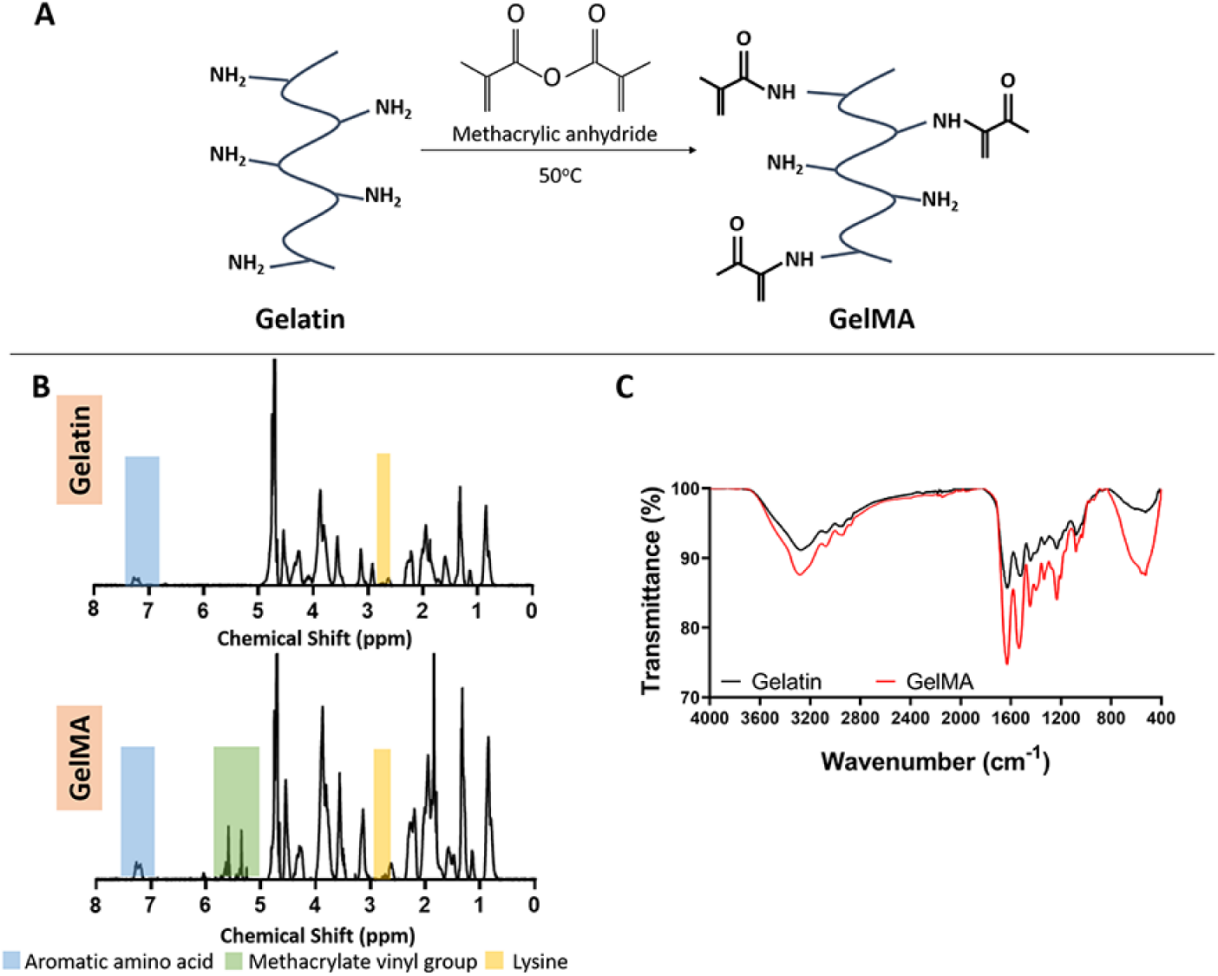
Synthesis and characterization of GelMA. (A) Schematic presentation of the methacrylation reaction of gelatin. (B) ^1^H-NMR and C) FTIR spectra of gelatin and GelMA.

Methacrylation of gelatin was also confirmed through ^1^H-NMR and FTIR analysis by using pristine and methacrylated gelatin (Figure 2). The ^1^H-NMR spectrum of GelMA displayed peaks at 7.3 ppm that belonged to the phenylalanine in gelatin, as well as new peaks at 5.5 and 5.3 ppm that were attributed to the methacrylate vinyl groups. The DM was calculated using the lysine methylene signal at 2.8–2.95 ppm for gelatin and GelMA, and it was found to be 85 ± 9%, which is quite high and in line with other studies in the literature.^38,39^ The FTIR spectra of gelatin and GelMA showed a peak at 3243 cm^–1^ because of N–H stretching. Furthermore, the methacrylation was demonstrated by peaks at approximately 1515 cm^–1^ and 1620 cm^–1^ that correspond to N–H bending and C=O stretching, respectively.^34^

In this study, the primary aim was to prepare PEtOx hydrogel through photo-cross-linking. In order to obtain a gel, the polymer with the highest DM required the shortest UV exposure. Since PEtOx-EI_70_-MA_36_ had the highest EI conversion and DM, it was selected to prepare PEtOx hydrogels. The resulting hydrogels were referred to as the POx-MA hydrogel throughout the rest of the article.

### 3.2 Microstructural Characterization

**Figure 3:**
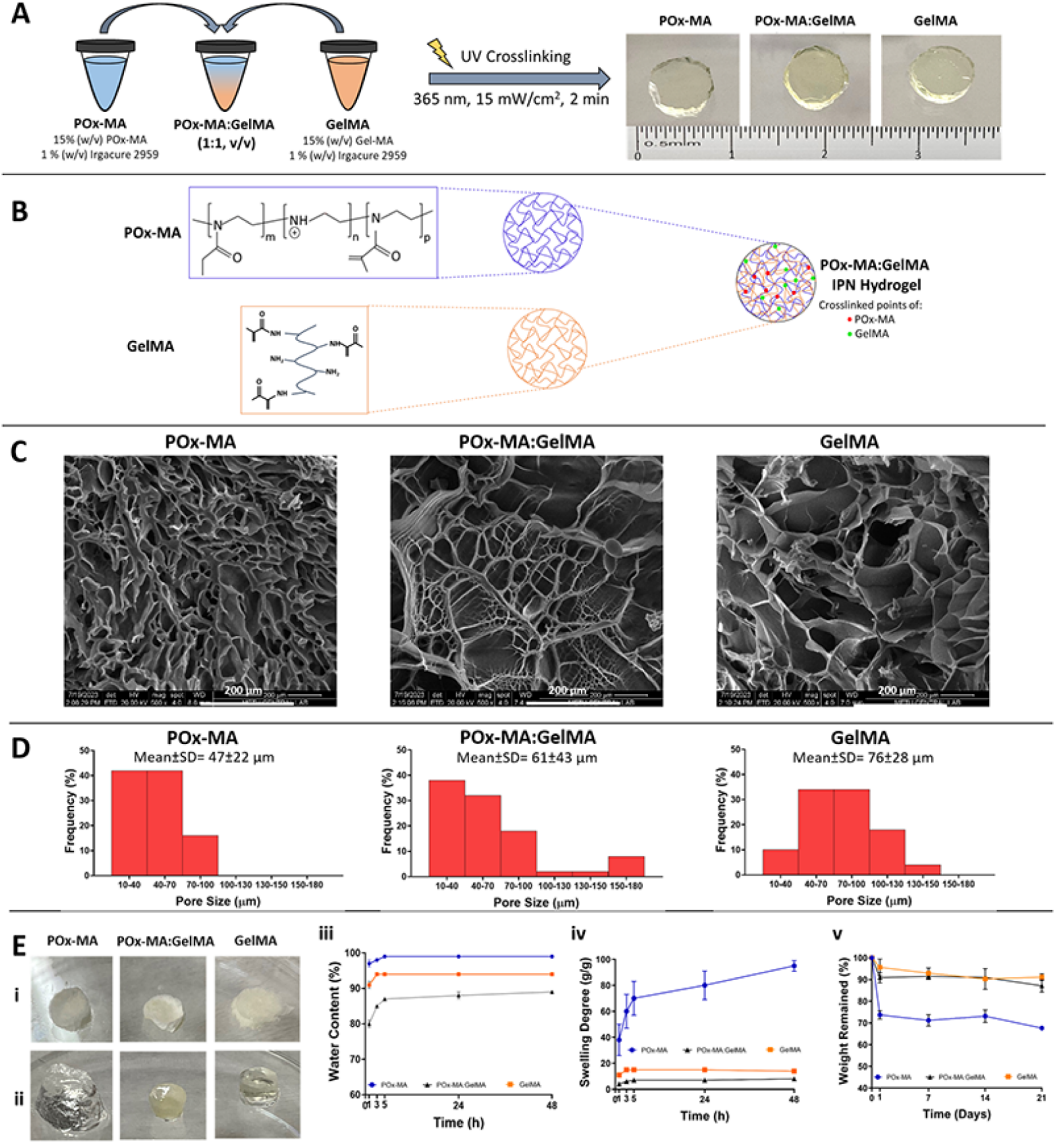
Hydrogel preparation and *in situ* characterization tests. (A) Schematic presentation of the preparation of the photo-cross-linked hydrogels. (B) Schematic illustration of POx-MA and GelMA polymer networks and IPN structure of the POx-MA:GelMA hydrogel. (C) SEM of the hydrogels. (D) Pore size distribution histograms obtained via NIH ImageJ pore size measurements using SEM micrographs of the hydrogels. (E) Photographs of (i) lyophilized and (ii) swollen hydrogels after incubation in distilled water for 48 h. (iii) water content (%) and (iv) swelling degree of the hydrogels immersed in distilled water at 37 °C for 48 h (*n* = 3). (v) *In situ* degradation of the hydrogels in PBS media (10 mM, pH 7.4) at 37 °C for 21 days (*n* = 3).

POx-MA, POx-MA:GelMA, and GelMA hydrogels were prepared by exposing the prepolymer solutions to UV light (365 nm, 15 W/cm^2^, 3 cm distance) for 2 min. All hydrogels were transparent after crosslinking (Figure 3A). Figure 3B shows the schematic illustration of POx-MA and GelMA polymer networks and the IPN structure of the POx-MA:GelMA hydrogel. SEM was used to study the microstructure of the cross-sections of the lyophilized hydrogels (Figure 3C). SEM examination revealed distinct pore structures, with POx-MA having small pores (47 ± 22 μm), GelMA showing larger pores (76 ± 28 μm), and POx-MA:GelMA displaying intermediate pore sizes (61 ± 43 μm) (Figure 3D). Pore size distributions of POx-MA were in the range 10–100 μm. POx-MA:GelMA and GelMA had similar pore size distributions 10–180 and 10–150 μm, respectively. The optimum pore size range was reported to be 20–120 μm for wound healing.^40^

### 3.3 Swelling Properties

The photographs of the lyophilized and swollen hydrogels after incubation in distilled water for 48 h are presented in Figure 3E-i and 3E-ii. Analysis showed that water absorbed by the hydrogels increased rapidly in the first 5 h and then practically stopped changing and reached equilibrium (Figure 3E-iii). The equilibrium water contents after 48 h were determined to be quite close, 99, 94, and 89% for POx-MA, GelMA, and POx-MA:GelMA, respectively. The equilibrium water content calculation did not show the difference in water absorption by the hydrogels very clearly; however, this difference was better represented upon determination of the swelling degrees, which is the amount of water the hydrogels absorbed per unit dry weight (Figure 3E-iv). The swelling degrees of POx-MA, GelMA, and POx-MA:GelMA were calculated to be 95, 14, and 8 g/g, respectively. Higher crosslinking density, which is related with DM, reduces water uptake.^41^ In this study, in order to prepare hydrogels, POx-MA and GelMA polymers were crosslinked through their methacrylate groups by UV crosslinking method. The degree of methacrylation of POx-MA and GelMA were 36% and 85%, respectively. Since the methacrylation degree of POx-MA was lower, which means it has less functional groups for crosslinking with UV light, the crosslinking density of POx-MA was lower compared to that of the GelMA hydrogel, resulting in a higher degree of swelling. The combination of two or more independent crosslinked networks, called interpenetrating network (IPN), facilitates the development of hydrogels with enhanced characteristics and functionality.^42^ IPN is the combination of two or more polymer networks into a single interconnected structure without the presence of covalent bonds. Within the architecture of an IPN, the constituent polymeric networks exhibit mutual entanglement resistant to physical separation, and both polymers are crosslinked.^43,44^ A semi-interpenetrating polymer network is characterized by the incorporation of two polymeric systems, wherein only one component exhibits cross-linked structural organization, while the second constituent maintains its linear macromolecular configuration. The linear polymer component is consequently physically entrapped within the three dimensional matrix of the crosslinked network.^17,45,46^ POx-MA:GelMA IPN hydrogels were produced by simultaneous UV crosslinking of POx-MA and GelMA polymers. POx-MA:GelMA IPN hydrogels had the lowest swelling degree (8 g/g). Probably they were more compact gels having higher crosslinking density because of the additional crosslinking of the second network and reducing their ability to swell. Water content of GelMA was found to be within the reported values (85–95%) in the literature.^47,48^ PEtOx is known for its superior hydrophilic character.^49^ In the literature, PEtOx based hydrogels have generally been developed by copolymerization starting from monomers or by grafting with monomer species. Acrylic acid is one of the most used molecules in order to functionalize PEtOx, and therefore to obtain a hydrogel via UV or gamma ray induced polymerization. For instance, Czich et al. prepared bis(acrylate) functionalized poly(2-ethyl-2-oxazoline)s (PEtOx-DA) by CROP to obtain hydrogels with 3D microstructures via two photon polymerization.^50^ They investigated the effect of chain length (degree of polymerization: 10 and 50) of PEtOx on the swelling property of the hydrogels. Their findings revealed that the water content of the hydrogels ranged from 50% to 90%, depending on the degree of polymerization of the PEtOx macro monomers and the crosslinking density. Additionally, they compared the swelling behavior of PEtOx-DA with a commercially available diacrylated poly(ethylene glycol) (PEG-DA) of a similar degree of polymerization (13 for PEG-DA vs 10 for PEtOx-DA) and showed that both exhibited comparable water retention, around 50%. Similarly, another study in the literature showed that poly(2-ethyl-2-oxazoline)/acrylic acid hydrogels had a swelling ratio of around 75% at pH 7.0.^51^ Unlike other studies, Nahm et al. functionalized PEtOx with furan and maleimide and fabricated hydrogels with 84% water content via melt electrowriting.^52^ POx-MA hydrogels fabricated in this study exhibited high EWC (99%) and swelling degree (94 g/g), which is comparable to those reported in the literature.

### 3.4 *In Situ* Degradation

Biodegradability is an important characteristic for tissue engineering scaffolds. The optimal degradation rate of a scaffold must correspond with the rate of new tissue formation, which differs according to the individual tissue undergoing regeneration. In this study, degradation tests were conducted in PBS (10 mM, pH 7.4) at 37 °C for 3 weeks. GelMA and POx-MA:GelMA demonstrated slower degradation, while POx-MA degraded significantly over 21 days (Figure 3E-v). The remaining weights after 3 weeks were found to be 91 ± 2%, 87 ± 3%, and 68 ± 1% for GelMA, POx-MA:GelMA, and POx-MA, respectively. Hydrolysis of the polymer backbone or crosslinks is the primary mechanism behind the degradation of hydrogels.^53^ POx-MA hydrogels had lower crosslinking density due to their low DM with respect to GelMA; therefore, they had a higher degree of swelling. Thus, the absorption of more water resulted in increased hydrolysis and degradation of POx-MA hydrogels. On the other hand, higher crosslinking density in GelMA increased the rigidity of the material and reduced the absorption of water required for hydrolysis and most likely decreasing the degradation rate. Meanwhile, there was no significant difference between GelMA and POx-MA:GelMA in terms of degradation rate, simply because of their similar water retention capacities. Photo-cross-linking is a practical technique that offers a number of benefits over chemical and physical crosslinking techniques, particularly with regard to enabling control over the structure’s stability, crosslinking duration, and mechanical strength.^54^ Thus, methacrylation is a commonly used approach to develop photo-crosslinkable hydrogels, as in our study. Benzophenone is another molecule that is also used for photo-cross-linking. For instance, Li et al. developed poly(2-ethyl-2-oxazoline)-benzophenone (PEtOx-BP) copolymer to prepare nanofiber structures via electrospinning.^31^ They reported a relatively high irradiation time (10 min) required to obtain a stable structure and noted that temperature and humidity also had a significant effect on the stability of the structures. In contrast to reported studies in the literature, methacrylation of PEtOx carried out in this study yielded effective and rapid crosslinking, where a stable hydrogel was obtained.

### 3.5 Compressive Mechanical Properties

**Figure 4:**
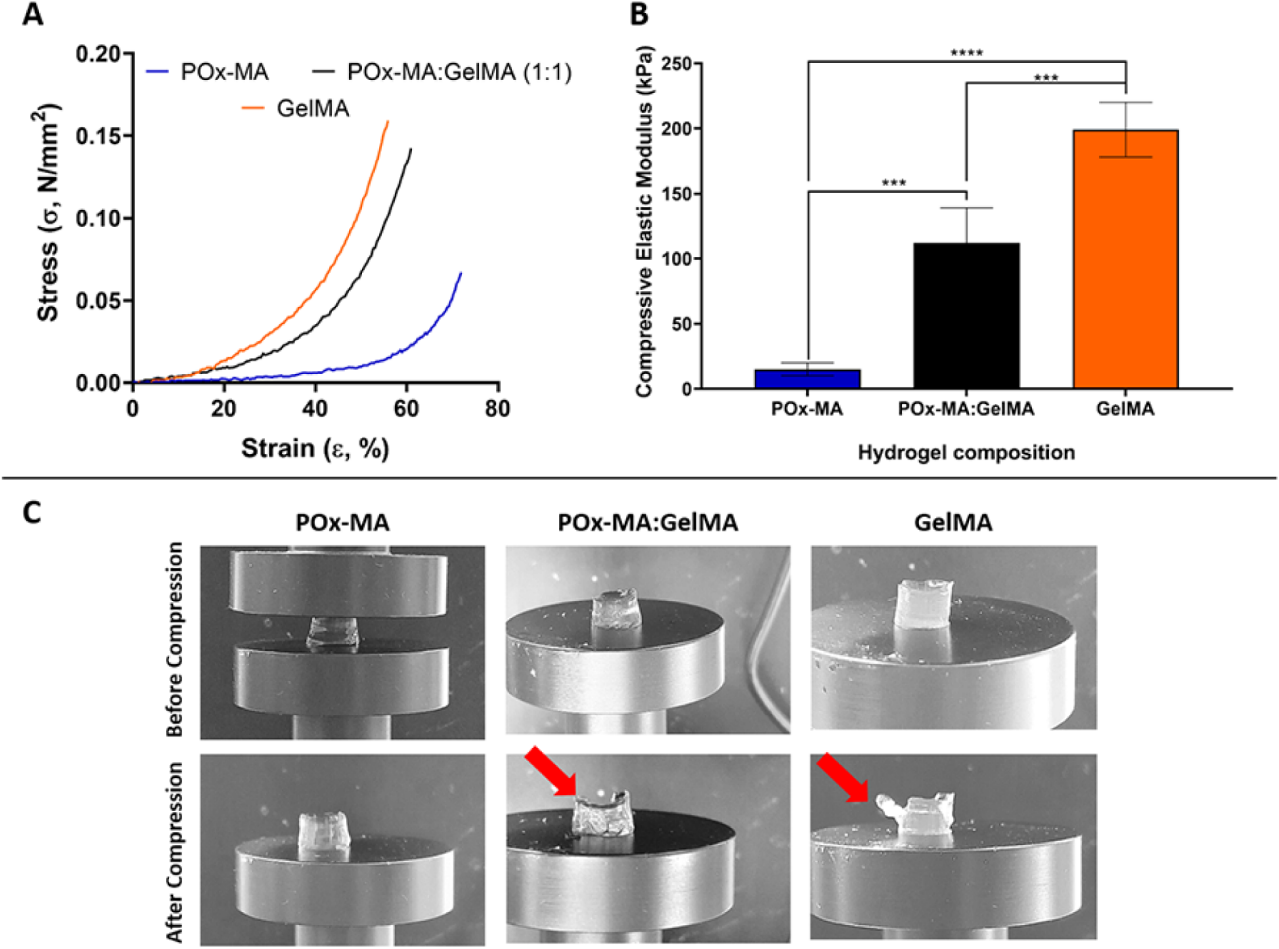
Compressive mechanical analysis of the hydrogels. (A) Stress–strain curves of the hydrogels (*n* = 5). (B) Compressive elastic moduli of the hydrogels calculated from the slope of linear region between 20% and 40% strain of the stress–strain graph. (C) Photographs of the hydrogels before and after compression test. Red arrows show the cracks occurred after compression. Statistical analysis was carried out by using one-way ANOVA. ***p < 0.005, ****p < 0.0001.

Compression tests were utilized to investigate the mechanical characteristics of the hydrogels. The stress-strain graphs of the samples were plotted, as shown in Figure 4A. At similar strain levels, GelMA shows the highest stress values, suggesting greater resistance to deformation. Meanwhile, the POx-MA hydrogel appears to be the least resistant to deformation, as evidenced by its lowest stress values within the same strain range. POx-MA:GelMA demonstrates intermediate mechanical properties, balancing the characteristics of both components. Figure 4B presents the bar graph that compares the compressive elastic moduli of the hydrogels, which were determined by the slope of the linear region’s between 20% and 40% strain on the stress–strain graph. GelMA shows the highest compressive elastic modulus (199 ± 21 kPa), signifying the greatest stiffness, while POx-MA exhibits the lowest compressive elastic modulus (15 ± 5 kPa), indicating it is the most flexible hydrogel. The POx-MA:GelMA hydrogel demonstrates a compressive modulus (112 ± 27 kPa) between these two values.

The deformation behavior of the hydrogels before and after compression is shown in Figure 4C. While GelMA and POx-MA:GelMA show significant deformation with cracks in their structure (indicated by red arrows), POx-MA exhibits the least deformation and excellent structural recovery. The data collectively demonstrate that GelMA has the highest mechanical strength, followed by the POx-MA:GelMA hydrogel, while POx-MA is the least robust material under compression. The POx-MA:GelMA hydrogel achieves a balance between an adequate degree of strength and flexibility. It has been reported that the degree of methacrylation, polymer concentration, and UV exposure time are important parameters that affect the mechanical properties of hydrogels.^55^ For instance, Schuurman et al. reported that it is possible to create GelMA hydrogels with compressive moduli ranging from 5 to 180 kPa by adjusting the GelMA (with a DM of 75 ± 9%) precursor solution’s concentration from 5% to 20%.^56^ In contrast to GelMA, POx-MA has a significantly lower DM, which results in lower mechanical strength, as expected. Nevertheless, the compressive elastic modulus of POx-MA (15 ± 5 kPa) still meets the mechanical requirements for soft tissue biomedical applications.^57^ The mechanical properties of POx-MA, as demonstrated in this study, align closely with the range of values reported for diacrylated PEG hydrogels (4.73 ± 0.5 and 503 ± 7.0 kPa) in the literature, highlighting its suitability as a potential substitute for PEG in biomedical applications.^58^ Furthermore, using POx-MA and GelMA together to develop an IPN hydrogel improved the mechanical strength, which implies the possibility of tuning the mechanical properties for desired biomedical applications.

### 3.6 *In Vitro* Cell Culture Studies

#### 3.6.1 Cell Viability, Proliferation, and Morphology on Hydrogels

**Figure 5:**
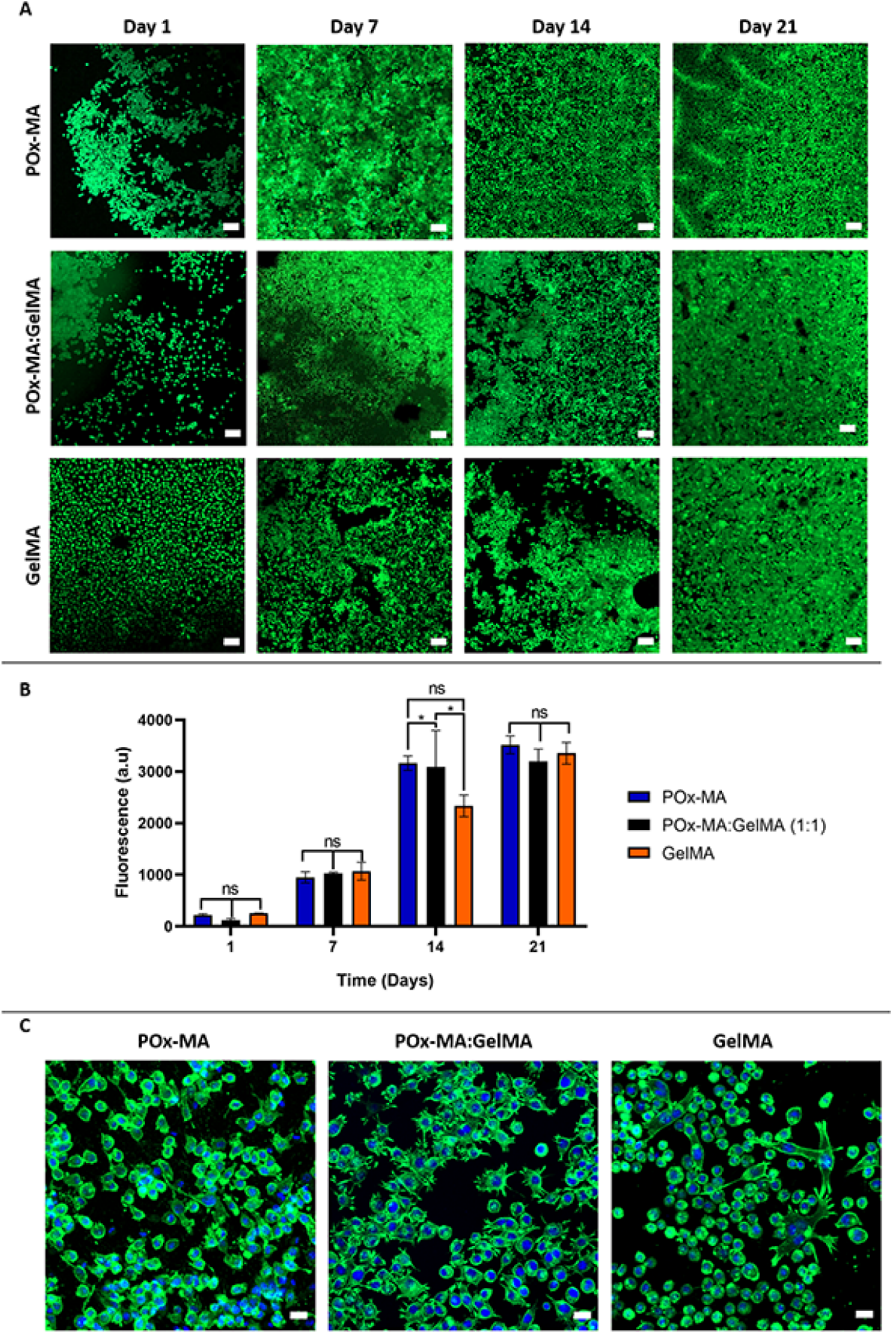
Cell viability and morphology of the hydrogels. (A) Confocal micrographs of Live/Dead staining (Initial cell seeding density: 3 × 10^4^ cells/scaffold). Scale bar: 100 μm. (B) Alamar Blue proliferation results on days 1, 7, 14, and 21. Statistical analysis was carried out using two-way ANOVA. *p < 0.1, and ns: not-significant. (C) Confocal micrographs of fibroblasts stained for cytoskeletal elements (Phalloidin, green in color) and nucleus (DAPI, blue in color). Scale bar: 20 μm.

The hydrogels were seeded with L929 cells, and their cytocompatibility was evaluated in terms of cell viability, proliferation, and morphology of the cells over 21 days. Wound healing is a complex process that typically takes 4 to 6 weeks, progressing through hemostasis, inflammatory, proliferative, and remodeling phases. Fibroblasts play a key role in ECM deposition, collagen synthesis, and wound contraction, particularly during the proliferative and remodeling phases, which begin at around day 4 and continue until the end of the healing process.^59^ Therefore, long-term fibroblast culturing on hydrogels (such as 21 days) is important to assess their cytocompatibility, stability, and ability to support continuous cell proliferation. Figure 5A displays the live/dead results of the POx-MA, POx-MA:GelMA, and GelMA hydrogels. According to the results obtained on day 1, all groups had similar initial cell viability. In the following days, all groups exhibited elevated fluorescence and cell density, which suggests cellular proliferation. However, on days 7 and 14, POx-MA and POx-MA:GelMA displayed higher cell distribution compared to GelMA, which implies that POx-MA supports better cell proliferation over time. Nevertheless, cell densities on POx-MA, POx-MA:GelMA, and GelMA hydrogels were similar on day 21.

Cells exhibited high viability, over 90%, across all groups. The Alamar Blue cell proliferation assay was conducted at different time points (1, 7, 14, and 21 days) for the three hydrogel types (Figure 5B). A general pattern of increasing fluorescence over time suggests that cell proliferation progressed during the 21 days of culture. Although there was a slight difference between POx-MA-containing groups and GelMA on day 14, fluorescence intensities depending on cellular metabolic activity, were not statistically different on day 21. Live/dead micrographs and Alamar Blue results suggest that all groups had roughly the same cellular density at the end of the incubation period. As shown in live/dead staining (Figure 5A) and Alamar Blue assays (Figure 5B), fibroblasts exhibited strong proliferation over 21 days, with high viability dominating the imaging field. Although natural cell turnover may occur, the intense green fluorescence in the live/dead images indicates a high density of viable cells, overshadowing red signals from dead cells. Overall, these results confirm that the hydrogels provide a stable and biocompatible microenvironment, effectively supporting long-term fibroblast growth and proliferation. Cell morphologies on the hydrogels were examined using DAPI and Phalloidin counterstaining at the end of the culture (on day 21). It was observed that the cells adhered well and elongated in all hydrogel groups. Meanwhile, cell filopodia were observed more densely on PEtOx-containing hydrogels (POx-MA and POx-MA:GelMA) compared to GelMA hydrogels, where cells exhibited a more rounded morphology. Overall, the results of *in vitro* cell culture experiments showed that POx-MA provided a supportive environment for cell proliferation and adherence. Moreover, the presence of POx-MA improved the bioactivity of GelMA. The biocompatibility and resemblance to natural ECM properties of GelMA are widely recognized. Numerous studies have demonstrated that the presence of cell-binding motifs, such as RGD sequences, in GelMA hydrogels provides an ideal environment for cell adhesion, proliferation, and differentiation.^60−62^ Although PAOx-based hydrogels have not been investigated as extensively as GelMA, PAOx is well-known for its potential as a nonfouling material and its biocompatibility. However, the literature suggests that PAOx-based materials generally lack natural cell adhesion sites, which can limit cellular attachment and proliferation. Therefore, PAOx-based materials incorporated with RGD sequences were developed to enhance cell attachment and proliferation.^28,63^

In this study, the POx-MA hydrogel showed great potential for cellular activity, although the cellular adhesion was quite similar to GelMA. The reason for improved cellular adherence on POx-MA is based on the presence of positively charged amine groups on the polymer backbone, which lead to electrostatic interactions with negatively charged cell membranes, as observed in cationic polymers published in the literature.^64,65^ Cell adhesion determines migration, proliferation, differentiation, and other critical cell behaviors and serves as the basis for communication between cells and their surroundings. However, the favorable effect of positively charged EI groups is limited by the amount of these moieties.^66^ For instance, Shan et al. reported that the viabilities of human embryonic kidney cells (HEK293) were 87%, 57%, and 3% for PEtOx-EI with 15%, 28%, and 53% EI units.^33^ However, they also showed that the cytotoxic effect of EI units was blocked by the methacrylation of these polymers, and cell viability was around 90% for PEtOx-EI_53_-MA_35_, which had similar EI conversion and DM to POx-MA used in our study.

Cell adhesion, viability, proliferation, and migration are regulated by interactions between cells and the extracellular matrix (ECM), which are influenced by the adsorption of adhesive proteins and the physicochemical properties of surfaces.^67^ In this study, POx-MA and GelMA hydrogels exhibited a gradual water absorption process, reaching a maximum swelling degree within 24 h, while cell adhesion occurred within the first 4 h, indicating that swelling did not negatively impact attachment. The presence of amine groups in POx-MA and RGD motifs in GelMA provided favorable conditions for cell adhesion. Additionally, *in situ* degradation experiments showed some weight loss within the first day but overall stability for 21 days, ensuring a supportive environment for cells. Moreover, the high swelling capacity of the hydrogels enhanced cell migration and proliferation by promoting nutrient and oxygen diffusion, making them suitable for long-term cell culture and tissue engineering applications.

#### 3.6.2 *In Vitro* Wound Healing Scratch Assay

**Figure 6:**
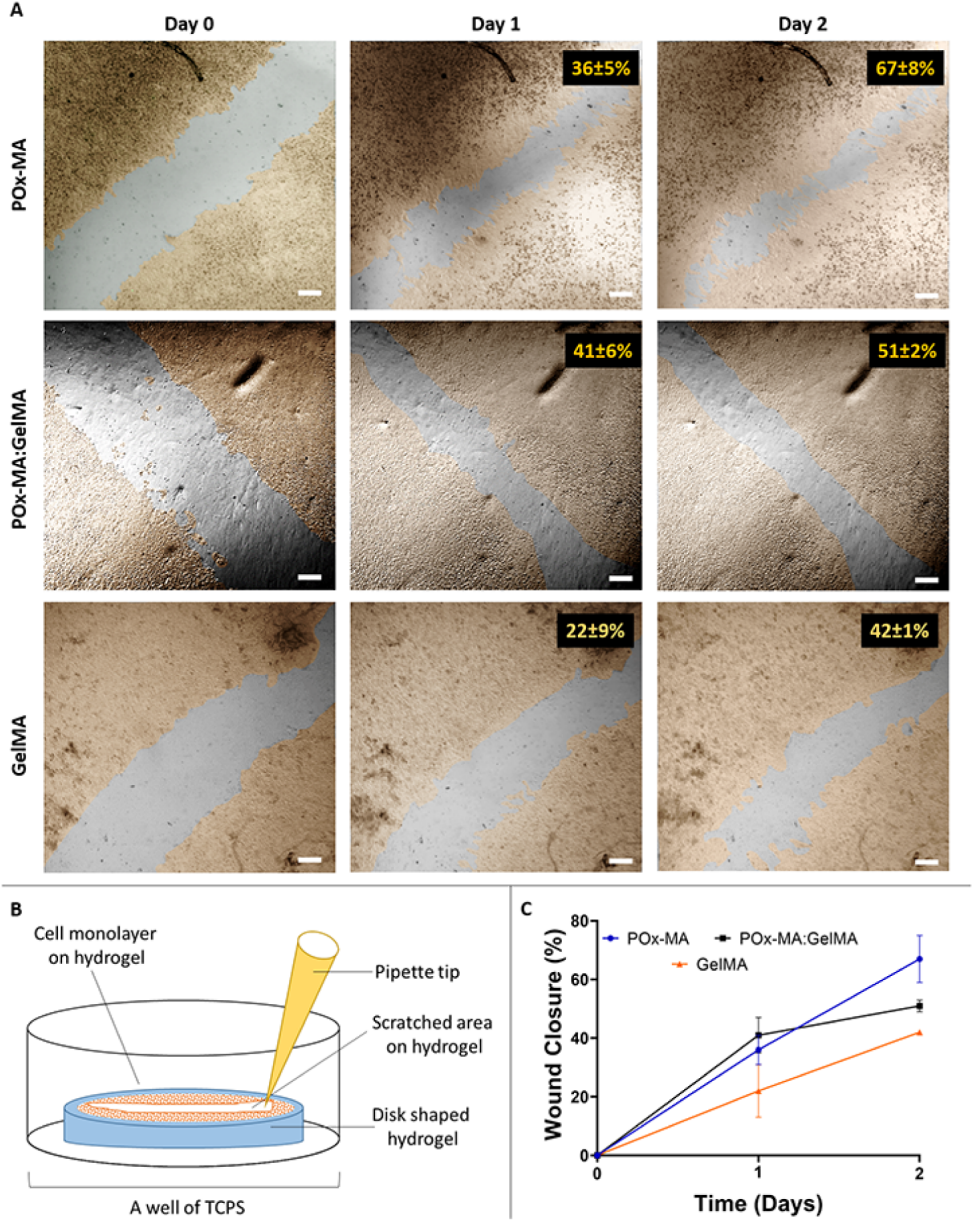
Wound healing scratch assay using L929 fibroblast cells. (A) Confocal micrographs of the wound healing scratch assay. Cells were pseudo colored using Adobe Photoshop software in order to obtain better visualization. (B) Schematic illustration of the scratch test on the hydrogels. (C) Wound closure (%) on the hydrogels.

In the process of wound healing, one of the most important phases is the migration of native cells from the wound boundary.^68^ The effect of the hydrogel composition on fibroblast migration was assessed via an *in vitro* wound healing scratch test. Figure 6 displays micrographs of scratches and wound closure (%). The results obtained on day 1 showed that the scratch wound closure on POx-MA:GelMA was significantly greater than on GelMA (*p<0.05). However, as time progressed, faster migration was observed for POx-MA, with 67 ± 8% wound closure. On the contrary, fibroblasts displayed slower migration on GelMA hydrogel, with 42 ± 1% wound closure. These results correlated with the morphological analysis (Figure 5C), where the cells were more adherent on the POx-MA and POx-MA:GelMA hydrogels. Thus, the cells have a better environment on POx-MA-containing hydrogels for spreading and migration, which also improves wound healing. The *in vitro* wound healing capacity of gelatin and GelMA has been reported either when used alone or in the presence of bioactive substances.^69−72^ However, this study, to the best of our knowledge, is the first to demonstrate the potential of PAOx-based materials for wound healing.

### 3.7 Antibacterial Activity

**Figure 7:**
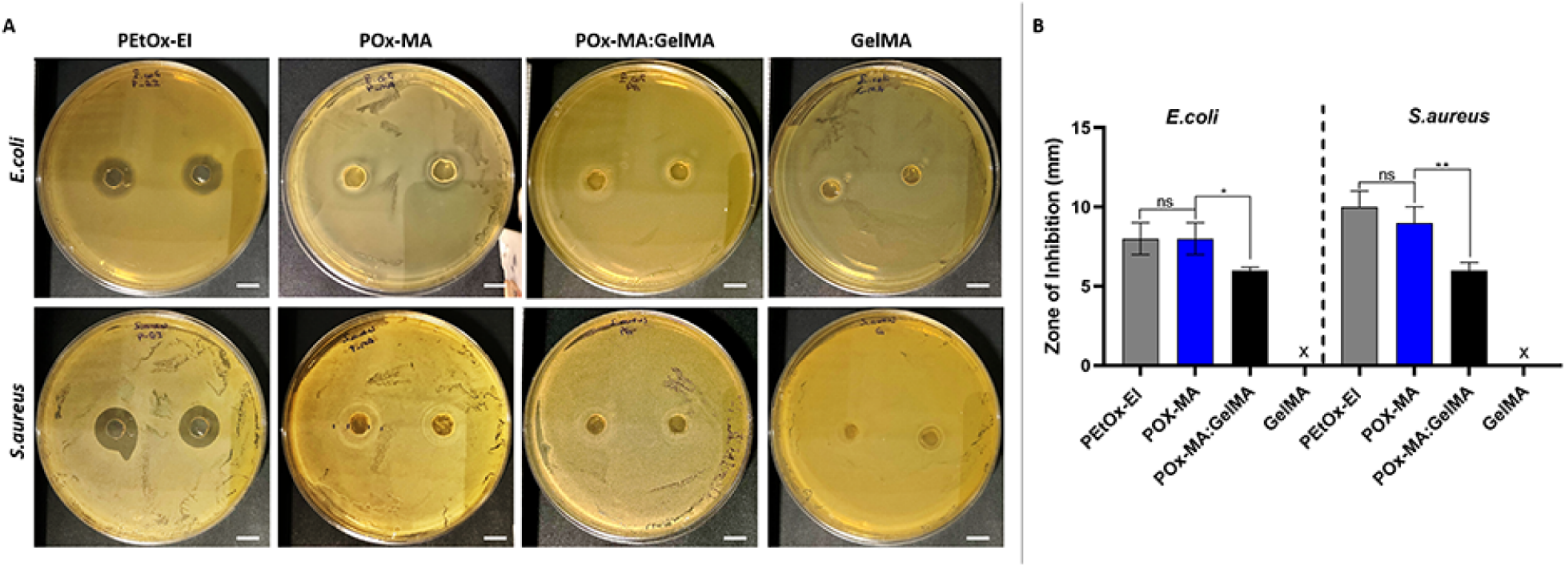
Antibacterial properties of the hydrogels against Gram-negative bacteria *E. coli* and Gram-positive bacteria *S. aureus*. (A) Images of inhibition zone and (B) graph showing the diameter of the zone of inhibition (in mm) of liquid PEtOx-EI, and the hydrogels. x: No inhibition zone. Scale bar: 5 mm.

The primary reason for prolonged wound healing and even worsening of wounds is bacterial infection. Materials with antibacterial properties prevent infection in wounds while also promoting the wound healing process.^73^ The antibacterial efficacy of the hydrogels against *E. coli* and *S. aureus* was assessed by using the zone of inhibition test (ZOI).^34,74^ The samples were cultured with microorganisms for 24 h on agar plates, and clear inhibitory zones were seen in the PEtOx-EI solution, POx-MA, and POx-MA:GelMA hydrogels (Figure 7A). Partially hydrolyzed PEtOx displayed antibacterial activity because of the positively charged amine groups on its backbone. Since the methacrylation of hydrolyzed PEtOx occurred through its amine groups, the amount of positively charged amine groups in the POx-MA hydrogel was less than that of PEtOx-EI. As seen in Figure 7B, the strongest antibacterial activity against *E. coli* and *S. aureus* was observed for PEtOx-EI, where ZOI values were 8 ± 1 mm for *E. coli* and 10 ± 1 mm for *S. aureus*. The POx-MA hydrogel showed moderate zones of inhibition (ZOI*_E.coli_*:8 ± 1 mm, ZOI*_S.aureus_*:9 ± 1 mm). This is most probably due to free amine groups present on the polymer, which were not methacrylated. Smaller zones of inhibition were observed for the POx-MA:GelMA hydrogel (ZOI*_E.coli_*:6 ± 1 mm, ZOI*_S.aureus_*:6 ± 1 mm) compared to PEtOx-EI and POx-MA, indicating a reduced antibacterial effect. Meanwhile, no zone of inhibition was observed in the GelMA group, since the GelMA hydrogel had no antibacterial effect. Kelly et al. reported the antibacterial effect of EI groups on partially hydrolyzed PEtOx (PEtOx-EI) against *E. coli*, *S. aureus*, *P. aeruginosa*, and *C. albicans*, noting that antibacterial activity increased with the degree of EI conversion.^75^ In the literature, natural antioxidants are frequently used for their antibacterial effects.^34,76,77^ However, in our study, we were able to achieve antibacterial activity solely through the hydrogel itself, without the need for any additional components. The mechanism of the antibacterial efficacy of positively charged polymers begins with electrostatic attraction to the negatively charged bacterial membrane phospholipids. Then, the stabilizing calcium layer around the bacterial membrane is repulsed, and phase separation of charged and uncharged lipids in the membrane occurs, which is followed by degradation of the membrane and cell lysis.^75^ Similarly, Wei et al. explained the antibacterial effect of cationic groups on polylysine, where electrostatic attraction leads to membrane damage and pore formation, followed by polylysine entering the bacteria through these pores, disrupting normal physiological metabolism, and leading to microbial cell death.^36^ In our study, *in situ* degradation tests revealed some weight loss on the first day in an aqueous medium, where the hydrogels were fully immersed. During the antibacterial tests conducted on agar plates, such an extent of degradation was unlikely to occur. In the meantime, one of the reasons for clear inhibitory zones might be the diffusion of small molecules (or even polymer fragments) spreading into the agar and inhibiting bacterial growth. Although the crosslinked polymer is not released, its electrostatic interactions can still influence bacteria in the surrounding area through localized ionic interactions. Additionally, the hydrogels may modify the microenvironment by altering ionic balance or surface interactions, creating conditions that are less favorable for bacterial growth in the nearby region. In the literature, similar non-leaching antibacterial materials, such as chitosan, polylysine, and poly(ethylene imine), have been shown to exhibit inhibition zones due to surface charge repulsion effects, further supporting the role of electrostatic interactions in antimicrobial activity.^78−80^

## 4 Conclusions

This study demonstrates the potential of POx-MA hydrogels as novel biomaterials for biomedical applications, especially in wound healing. POx-MA displayed significant advantages due to its superior swelling capacity, flexibility, cell attachment ability, and inherent antibacterial properties. The development of an IPN that combined POx-MA and GelMA further improved these hydrogels’ mechanical strength and adaptability, allowing for the design of systems particularly suited to specific biomedical requirements. POx-MA and POx-MA:GelMA IPN structures provide a flexible and efficient platform for tissue engineering and wound care due to their superior wound closure capability, improved cell viability, and enhanced cell-material interactions, made possible by the free amine groups of POx-MA. Although this study showed how POx-MA hydrogel can aid in wound healing, its advantageous physicochemical and biological characteristics also suggest that it could find wider use in other areas of soft tissue engineering. Furthermore, the biocompatibility, chemical versatility, and robustness of POx-MA and POx-MA:GelMA IPN hydrogels highlight their potential as bioinks for three-dimensional (3D) bioprinting. These findings provide valuable insights into the development of next-generation hydrogel systems, which open up opportunities for biofabrication, soft tissue repair, and regenerative medicine.
